# Role of Canonical Wnt/β-Catenin Pathway in Regulating Chondrocytic Hypertrophy in Mesenchymal Stem Cell-Based Cartilage Tissue Engineering

**DOI:** 10.3389/fcell.2022.812081

**Published:** 2022-01-24

**Authors:** Xueqi Wang, Yiming Guan, Shiyu Xiang, Karen L. Clark, Peter G. Alexander, Lauren E. Simonian, Yuhao Deng, Hang Lin

**Affiliations:** ^1^ Department of Nephrology, Beijing Friendship Hospital, Capital Medical University, Beijing, China; ^2^ Department of Radiology, Fudan University Shanghai Cancer Center, Shanghai, China; ^3^ Department of Orthopaedic Surgery, University of Pittsburgh School of Medicine, Pittsburgh, PA, United States; ^4^ McGowan Institute for Regenerative Medicine, University of Pittsburgh School of Medicine, Pittsburgh, PA, United States; ^5^ Department of Orthopedic Surgery, Shanghai Sixth People’s Hospital, Shanghai Jiaotong University, Shanghai, China

**Keywords:** mesenchymal stem cells, hyaline cartilage, chondrocytic hypertrophy, chondrogenesis, cartilage tissue engineering

## Abstract

In the past 3 decades, the cartilage repair potential of mesenchymal stromal cells, or mesenchymal stem cells (MSCs), has been widely examined in animal studies. Unfortunately, the phenotype and physical properties of MSC-derived cartilage tissue are not comparable to native hyaline cartilage. In particular, chondrocytic hypertrophy, a phenotype that is not observed in healthy hyaline cartilage, is concomitant with MSC chondrogenesis. Given that hypertrophic chondrocytes potentially undergo apoptosis or convert into osteoblasts, this undesired phenotype needs to be prevented or minimized before MSCs can be used to repair cartilage injuries in the clinic. In this review, we first provide an overview of chondrocytic hypertrophy and briefly summarize current methods for suppressing hypertrophy in MSC-derived cartilage. We then highlight recent progress on modulating the canonical Wnt/β-catenin pathway for inhibiting hypertrophy. Specially, we discuss the potential crosstalk between Wnt/β-catenin with other pathways in regulating hypertrophy. Lastly, we explore future perspectives to further understand the role of Wnt/β-catenin in chondrocytic hypertrophy.

## Introduction

Articular cartilage, an essential component of the joint organ, helps maintain stability, distribute force and reduce friction during physical activity. Once damaged by trauma, disease, or aging, it is unable to fully self-heal due to its avascular nature (Shi et al., 2016). Consequently, focal cartilage defects may gradually destroy adjacent cartilage and negatively influence other joint components, eventually leading to the onset of osteoarthritis (OA) (Chen et al., 2006; Deng et al., 2019b).

Currently, several strategies are clinically used to treat focal cartilage defects, including autograft and allograft transplantation, microfracture, and autologous chondrocyte transplantation (ACI) (Chen et al., 2015). However, each of these techniques has its own limitations (Ahmed and Hincke, 2010). For example, autograft transplantation relies on the transfer of osteochondral tissue from a non-weight-bearing or lesser weight-bearing region to the defective area, for which the donor tissue may not be mechanically suited (Kon et al., 2013). The use of allografts may induce immunological rejection (Bugbee et al., 2012). The core technology of microfracture is drilling into the subchondral bone to release bone marrow, allowing marrow-derived stem cells to migrate into the defective area to facilitate the healing process. Unfortunately, the neo-cartilage generated after microfracture consists mainly of fibrocartilage, not hyaline cartilage (Mithoefer et al., 2009). Consequently, the neo-cartilage degrades in several years. ACI involves the extraction of chondrocytes from a non-weight-bearing area, expansion of the cells *in vitro*, and re-introduction into the defect area. This technique eliminates the possibility of an immune response, but the proliferation process is always accompanied by the loss of chondrocytic phenotype and function, known as dedifferentiation (Kon et al., 2009). In addition, the quality control for chondrocytes before implantation has not been established, which likely results in highly variable reparative outcomes (Pietschmann et al., 2012).

In the past 3 decades, the chondroinduction of mesenchymal stem/stromal cells (MSCs) has become a popular method to generate cartilage tissue for the repair of cartilage injury in animal models (Caplan, 1991). Furthermore, some clinical trials have shown the potential of MSC-based cartilage tissue engineering in the repair of chondral defects in humans (Gupta et al., 2016; Park et al., 2017; Qiao et al., 2020), but a greater sample size is required to confirm the reparative results and phenotype of newly formed cartilage. In spite of this exciting progress, extensive *in vitro* data and animal studies have shown that MSC-derived cartilage does not fully recapitulate the structure or composition of hyaline cartilage matrix (Bian et al., 2013; Watts et al., 2013; Yang et al., 2018). In particular, chondrocytic hypertrophy-relevant molecules, such as collagen type X (COL10) and Indian hedgehog (IHH), are found to be highly expressed in MSCs-derived cartilage (Chen et al., 2015), which are not observed in healthy hyaline cartilage. This undesired phenotype represents one of the major obstacles to the clinical application of MSCs for repairing articular cartilage. In this review, we first provide an overview of hypertrophy in MSC-derived cartilage, and briefly summarize current methods used in suppressing hypertrophy. Second, we focus on recent progress in the modulation of the canonical Wnt/β-catenin pathway for inhibiting hypertrophy. Lastly, we discuss the crosstalk between Wnt/β-catenin and other pathways in regulating hypertrophy.

### Hypertrophic Chondrocytes in Growth Plate

**FIGURE 1:**
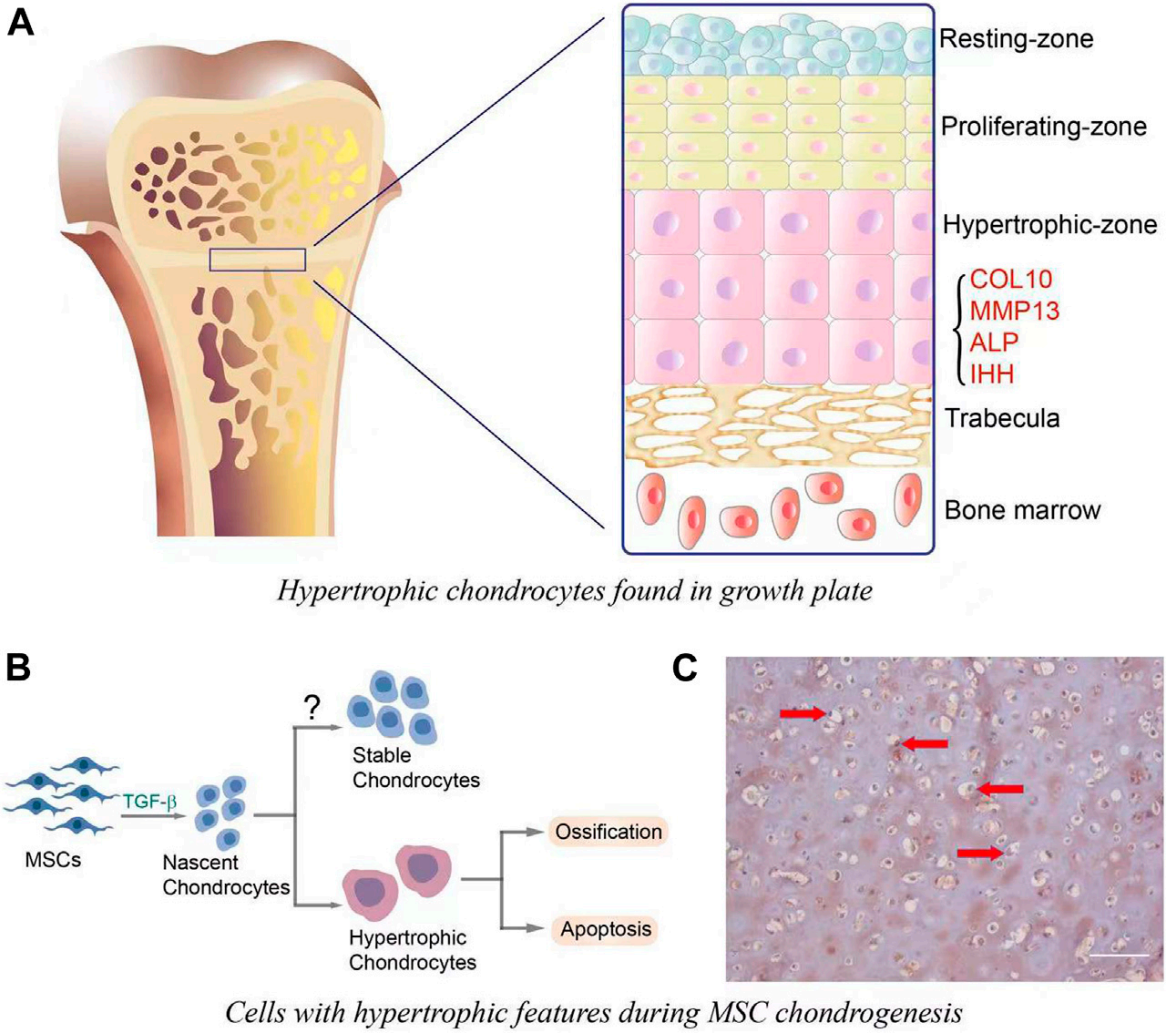
**(A)**. Hypertrophic chondrocytes observed in growth plate. Cells in the resting-zone, located at the end of bone, are round and small. Proliferating zone is adjacent to the resting-zone, and chondrocytes in this zone have the ability to proliferate and differentiate. The differentiation is also accompanied by the increase of cell size and the acquisition of hypertrophic phenotype, which is mainly characterized by the expression of hypertrophic markers, such as COL10, MMP-13, ALP, and IHH. **(B)**. *In vitro* MSC chondrogenesis and hypertrophic transition. In the initiation stage (∼7 days) of chondrogenesis, MSCs are differentiated into nascent chondrocytes, which then undergo hypertrophy and the following ossification or apoptosis. It is important to note that nascent MSCs-derived chondrocytes may have the potential to become stable chondrocytes, yet this has not been completely achieved. **(C)**. COL10 immunostaining for MSCs-laden hyaluronic acid (HA) construct that was subjected to 21 days of chondrogenic culture. The hypertrophic chondrocytes were characterized by large cell volume and the deposition of COL10 (brown staining, red arrows). Scale bar = 100 μm.

During endochondral ossification in skeletal development, several sequential events are observed, including cellular condensation of mesenchymal precursor cells, chondrogenic differentiation, hypertrophy and vascularization/osteogenesis (Sun and Beier, 2014). These stages are also clearly observed within the fetal growth plate, in which resting zone, proliferating zone, hypertrophic zone and osteogenic zones are clearly and simultaneously observed (Figure 1A). Hypertrophic chondrocytes contribute to skeletal development by increasing cell volume and tissue size. These cells also remodel the matrix to support osteogenesis, which is mainly characterized by the expression of runt-related transcription factor 2 (RUNX2), matrix metalloproteinase (MMP)-13, COL10, alkaline phosphatase (ALP) and osteogenic growth factors, such as bone morphogenetic protein (BMPs), IHH, and vascular endothelial growth factor (VEGF) among others (Sun and Beier, 2014).

### Cellular Hypertrophy in MSC Chondrogenesis

When MSCs are induced to undergo chondrogenesis with medium containing transforming growth factor (TGF-β), the newly formed cartilage recapitulates many features observed in hypertrophic chondrocytes in bone development, such as large cell size and high expression of hypertrophy-relevant markers (Chen et al., 2015) (Figures 1B,C). Therefore, the term “hypertrophy” was adapted to describe the phenotype of chondroinduced MSCs. Of note, the health states of donors and tissue sources affect MSC hypertrophy levels in both naïve and chondroinduced conditions. For example, the basal COL10 protein level in MSCs from donors with osteoarthritis (OA) was systematically (4.9 ± 1.2-fold) higher than that in normal MSCs (Mwale et al., 2010). After chondroinduction, articular cartilage-derived MSCs from osteoarthritis patients (OA-MSC) displayed higher expression of *RUNX2* when compared to the cells cultured in the growth medium (Hu et al., 2019). In comparison, the level of *RUNX2* in MSCs from bone marrow (BMSCs) did not change after chondrogenic culture. Moreover, the peak of *COL10A1* expression in chondroinduced OA-MSCs appeared earlier (on day 7) than BMSCs (on day 14), implying their intrinsic difference in hypertrophy potential (Liu et al., 2020). Therefore, the selection of MSC for cartilage repair requires careful considerations, which may significantly influence the quality of regenerated cartilage.

Currently, the mechanistic or physiologic sameness of *in vivo* and *in vitro* hypertrophy is not clear. A comprehensive and in-depth comparison of hypertrophy between these two processes is lacking. It has been demonstrated that hypertrophic transition in bone development is spatiotemporally regulated. For instance, IHH, released by hypertrophic chondrocytes, stimulates the production of Parathyroid hormone-related peptide (PTHrP), which not only induces resting chondrocytes into proliferating chondrocytes, but also negatively suppresses hypertrophy (Kronenberg, 2003). Panexxin3 and CCAAT enhancer binding protein beta (C/EBPβ), generated by pre-hypertrophic chondrocytes, and plays an essential role in promoting the transition of proliferative chondrocytes to hypertrophic chondrocytes (Marino, 2011). This well-orchestrated regulation and feedback network does not exist in MSC-derived chondrogenesis and hypertrophic transition.

As of now, it is not clear if MSCs can form stable chondrocytes with features similar to cells in hyaline cartilage. Many studies have shown that the hypertrophic chondrocytes derived form TGFβ-stimulated MSCs undergo ossification and apoptosis when tested in murine subcutaneous or intramuscular implantation experiments (Mueller and Tuan, 2008; Bian et al., 2013; Lee et al., 2013; Zhu et al., 2017). However, whether intraarticularly implanted MSCs-derived chondrocytes also undergo osteogenesis and/or apoptosis in humans is not conclusively known.

Currently, the exact mechanism underlying hypertrophy is not clear. Based on current evidence, it is hypothesized that chondrocytic hypertrophy is associated with high level of RUNX2 and activation of Smad1/5 signaling upon TGF-β treatment (Mueller and Tuan, 2008; Chen et al., 2015). Several pathways, including the BMP, TGF-β, fibroblast growth factor (FGF) and wingless-related integration site (Wnt) pathways (Yang et al., 2001; Yu et al., 2010; Shen et al., 2013; Wang et al., 2014; Zhong et al., 2015), were shown to regulate this process. In particular, low TGFβ type I receptor/BMP type I receptor ratio was shown to account for the high hypertrophy potential of naïve OA-MSCs (Liu et al., 2020). In the past two decades, numerous strategies have been tested to suppress hypertrophy during MSC chondrogenesis. For example, supplementing fibroblast growth factor (FGF)-9 or 18 after the initiation of chondrogenesis (≥14 days) suppressed hypertrophy-related changes, which functioned through FGF receptor-3 (Correa et al., 2015). Browe et al., found that hypoxia culture attenuated the hypertrophy phenotype during MSCs chondrogenesis through modulating the parathyroid hormone-related peptide (PTHrP)-Myocyte Enhancer Factor 2C (MEF2C) pathway (Browe et al., 2019). Curcumin also suppressed MSC hypertrophy through inhibiting IHH and Notch signaling (Cao et al., 2017). Among these methods, treatments that suppress Wnt/β-catenin signaling pathway have showed the most promising results in reducing hypertrophy level (Yang et al., 2012; Narcisi et al., 2015; Zhong et al., 2016), which are specifically discussed in this review.

### Canonical Wnt/β-Catenin Pathway in Chondrogenesis

The expansion of MSCs is partially driven by the Wnt pathway, which also maintains their potential (ten Berge et al., 2008; Cooper et al., 2011). Of note, the regulation of Wnt signaling depends not only on the presence of ligands but also on the action of endogenous antagonists of Wnt signaling (Usami et al., 2016). Numerous studies have shown that β-catenin-mediated canonical Wnt signaling exhibits the suppression of chondrogenesis (Day et al., 2005; Dong et al., 2007; Churchman et al., 2012; Wang et al., 2021). However, Deng et al. showed that *de novo* inhibiting β-catenin with XAV939 throughout a 21-days MSC chondrogenic pellet culture completely blocked the deposition of cartilage matrix and expression of chondrogenic markers (Deng et al., 2019a). Therefore, the exact role of the canonical Wnt/β-catenin pathway in MSCs chondrogenesis needs further investigation.

### Inhibition of Hypertrophy Through Modulating Wnt/β-Catenin Pathway

Extensive evidence has shown that Wnt pathway participates in chondrocytic hypertrophy during MSC chondrogenesis. For instance, the study from Yang et al. found that transient stimulation of the Wnt pathway promoted the expression of hypertrophic phenotype during the chondrogenic differentiation of MSCs in pellet culture (Yang et al., 2012). Melatonin induced chondrogenic hypertrophy, which resulted from upregulated Wnt/β-catenin signaling (Wang et al., 2021). Interestingly, the basal level of β-catenin protein in BMSCs isolated from OA patients was also significantly higher than BMSCs from healthy donors (Tornero-Esteban et al., 2015), suggesting Wnt/β-catenin may also account for the high hypertrophy level in native OA MSCs. However, the relevant studies have not been reported yet.

In contrast, inhibiting Wnt signaling with IWP2 (Narcisi et al., 2015; Diederichs et al., 2019b), DKK1 (Rojas et al., 2019), XAV939 (Deng et al., 2019a; Wang et al., 2021), PKF (Huang et al., 2018) prevented hypertrophic maturation of MSC-derived cartilage. It is important to note that the timing of introducing Wnt inhibitors needs to be carefully considered. Deng et al. found that the addition of XAV939 from the beginning of chondrogenic culture blocked both hypertrophy and chondrogenesis (Deng et al., 2019a). In this study, MSCs were encapsulated in hyaluronic acid-based scaffolds, so it is not clear whether such a phenomenon translates to other culture systems.

Regarding the mechanism, there are not too many relevant studies. The study by Deng et al. demonstrated that XAV939 reduced hypertrophy level by inhibiting the Smad1/5-RUNX2 pathway (Deng et al., 2019a). In another study, IWP-2 was found to suppress hypertrophy through inhibiting the gene expression of *BMP7* and *Gli1,* which are associated with BMP and IHH pathways (Diederichs et al., 2019b). Dreher et al. also reported a reduction in MSC-derived chondrocyte hypertrophy following IWP-2 inhibition of Wnt signaling. However, the mechanism discovered was different (Dreher et al., 2020). Specifically, IWP-2 was shown to inhibit hypertrophy through decreasing levels of myocyte enhancer factor 2C (MEF2C) and RUNX3. Interestingly, this study also showed that the absence of RUNX2 did not prevent hypertrophy. Therefore, Wnt inhibitors suppress hypertrophy through different mechanisms, which highly depend on their respective direct target(s). The resulting inhibition is also dependent on the cell types and culture platforms, such as two-dimensional versus three-dimensional cultures, and pellet versus scaffold cultures.

### Interaction of Wnt/β-Catenin Signaling With Other Pathways in Hypertrophy

Currently, the network between Wnt signaling and other pathways during hypertrophy has not been established. In this review, we would like to summarize current findings from different studies and map the network. In the study of Bouaziz et al., it was found that the hypoxia-inducible factor 1α (HIF1α)-β-catenin interaction was a negative regulator of Wnt signaling and MMP-13 transcription (Bouaziz et al., 2016). Co-transfection of lymphoid enhancer binding factor 1 (LEF1) and β-catenin in chicken sternal chondrocytes induced hypertrophy through activating RUNX2, which was inhibited by high-mobility group box 2 (HMGB2) (Taniguchi et al., 2011). Zhu et al. found that downregulation of α-B-crystallin (CRYAB) reduced RUNX2 level through canonical Wnt/β-catenin pathway, resulting in a lower potential level of hypertrophy/osteogenesis. They also determined that CRYAB can interact with β-catenin and protect it from ubiquitination and degradation (Zhu et al., 2020). Recently, Riedl et al. reported that MSC-based pellet cultures underwent hypertrophy when retinoic acid receptor (RAR) pathway was activated. As a result of RAR activation, there was increased expression of Wnt2b and Wnt5a, which subsequently increased Wnt/β-catenin pathway activity (Riedl et al., 2020).

Yes-associated protein (YAP) and transcriptional coactivator with PDZ-binding motif (TAZ) signaling are important in the development of cartilage (Vanyai et al., 2020). Specifically, low level of yes-associated protein (YAP), a transcription factor of the Hippo pathway, was associated with the formation of articular cartilage from MSCs (Lee et al., 2020). Using murine models, Deng et al. demonstrated that *Yap1* overexpression significantly reduced *COL10A1* level, which however was at the expense of reduced chondrogenesis. In contrast, suppressing YAP with siRNA promoted the expression of both *COL2A1* and *COL10A1* without selection (Deng et al., 2016). Recently, mechanical loading, under certain conditions, was shown to suppress hypertrophy (Aisenbrey et al., 2019). In the study by Lee et al., MSCs cultured in 3D chondrogenic combinatorial system with 40% strain showed a trend toward hypertrophic chondrocytes, indicated by the high level of RUNX2, which was also accompanied with more YAP expression (Lee et al., 2020). Of note, the association between mechanical loading and Wnt/β-catenin has been previously demonstrated (Sen et al., 2011; He et al., 2020). These observations raise the question, how does the YAP pathway interact with Wnt/β-catenin in hypertrophy? To the best of our knowledge, no relevant study has been published that directly addresses this question. However, interactions between YAP and Wnt/β-catenin have been previously explored in other cell types. In studies of colon cancer, the inhibition of Wnt signaling suppressed the expression of YAP. Conversely, YAP could be upregulated by activating Wnt pathway (Konsavage et al., 2012; Park et al., 2015). During the fibrosis process, stimulation of Wnt pathway caused dissociation of YAP from β-catenin destruction complex after they translocate to nucleus and modulate downstream target genes (Piersma et al., 2015). Unfortunately, the Wnt/β-catenin pathway was not simultaneously investigated in this study. Based on the findings above, we speculate that Wnt/β-catenin is upstream of the YAP pathway in regulating chondrocytic hypertrophy, which should be further explored in the future.

**FIGURE 2:**
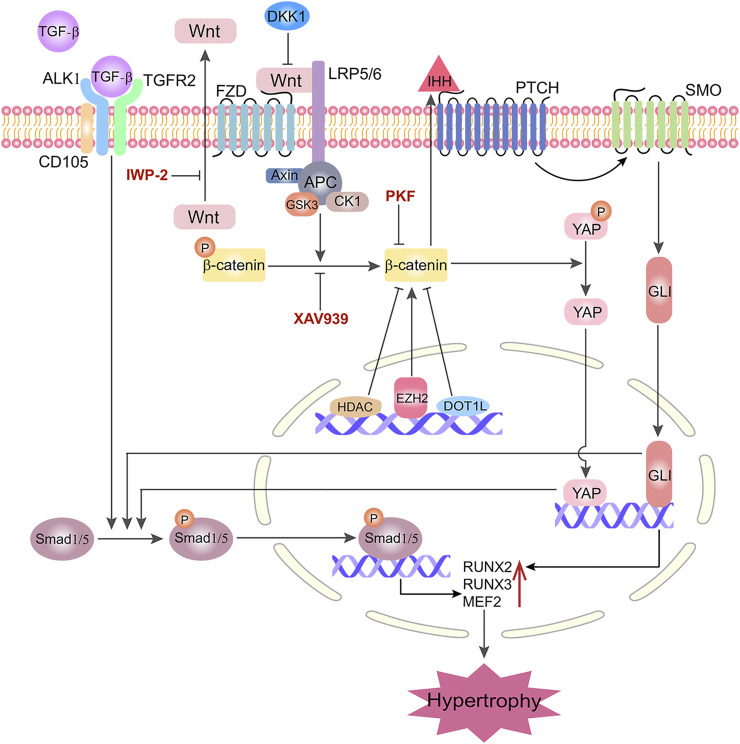
Interactions of Wnt/β-catenin signaling with other molecules/pathways in governing chondrogenic hypertrophy in MSC chondrogenesis. LRP5/6: Lipoprotein Receptor-Related Protein 5/6; FZD: Frizzled; ALK1: Activin Receptor-Like kinase 1; CD105: Endoglin; TGFR2: TGF-Receptor 2; PTCH: Patched; SMO: Smoothened; GlI: Cubitus interruptus; MEF2: Myocyte enhancer factor; DC: Destruction Complex; APC: Adenomatous Polyposis Coli; CK1: Casein kinase 1; GSK3: Glycogen synthase kinase 3; EZH2: Zeste homologue 2; DOT1L: Disruptor of telomeric silencing 1-like; HDAC4: Histone deacetylase 4; IHH: Indian Hedgehog; YAP: Yes-associated protein; SIRT1: Silent mating type information regulator 2 homolog 1; XAV939, IWP-2, PKF and DKK1 are representative Wnt/β-catenin inhibitors in suppressing hypertrophy.

The molecules related to histone modification were also shown to participate in hypertrophy through Wnt pathways. Cornelis et al. found that Disruptor of telomeric silencing 1-like (Dot1L)-deficient mice could present Wnt signaling hyper-activation and ectopic chondrocyte hypertrophy (Cornelis et al., 2019). The study by Chen et al. demonstrated that inhibition of histone methyltransferase enhancer of zeste homologue 2 (EZH2) inhibited *IHH*, *MMP-13*, *ADAMTS-5* and *COL10A1* expression, thus ameliorating OA development (Chen et al., 2016). Interestingly, this study also showed that inhibition of EZH2 silenced β-catenin signaling. A genome-wide analysis has shown that the Wnt/β-catenin transcriptome was inhibited after regulating of HDAC (Chen et al., 2011). Moreover, in the study of Smith et al., the Wnt-related pathway’s transcription levels were restricted by overexpression of HDAC (Smith and Frenkel, 2005). In light of these findings, we summarized the potential interaction of Wnt pathways and other molecules and pathways controlling hypertrophy in Figure 2. In brief, when TGF-β binds to its receptor Activin-receptor like kinase (ALK) 1 accompanied with its co-receptor CD105, it phosphorylates Smad1/5 and stimulates the transcription of RUNX2, RUNX3 and MEF2, initiating the hypertrophy process. The activation of Smad1/5 is also regulated by Wnt-β-catenin, YAP (Gabriel et al., 2016) and IHH (Handorf et al., 2015). Particularly, the Wnt/β-catenin pathway plays an essential role in regulating chondrocytic hypertrophy, which is activated by EZH2 and inhibited by DOT1L, HDAC4, DKK1. XAV939 and other β-catenin inhibitors, such as IWP-2 and PKF, eventually inhibit Smad1/5, IHH and YAP, which collectively reduce chondrocytic hypertrophy.

## Conclusion and Future Perspectives

Chondrocytic hypertrophy remains a crucial obstacle when developing MSC-based therapy for the repair of hyaline cartilage injury. The hypertrophic phenotype in MSC-derived cartilage is most identified and characterized *in vitro*, or in animal models that are often used to assess bone formation, such as subcutaneous or intramuscular implantation. Whether MSC-derived chondrocytes also maintain hypertrophic features after long-term intraarticular implantation in humans is not clear. Of note, the concentration of active TGF-β in native synovial is around 60 pg/ml (de Sousa et al., 2019), which is significantly lower than that are used in inducing MSC chondrogenesis *in vitro* (1–10 ng/ml). In addition, the hypoxic environment found in the knee joint may also suppress hypertrophy. Therefore, the potential to generate hyaline cartilage from MSCs within native joints is still possible, which however needs to be validated in clinical studies.

The critical role of Wnt/β-catenin in promoting hypertrophy has been reviewed above. Largely due to our limited knowledge about the pathways controlling hypertrophy, the molecular mediation of Wnt and hypertrophic gene expression need further study. Current evidence suggests that RUNX2 may account for the hypertrophy phenotype. However, knocking down RUNX2 did not entirely suppress *COL10A1* expression (Chen et al., 2020), which suggests that RUNX2 may not be the only transcriptional factor controlling *COL10A1* expression (Dreher et al., 2020). As described above, activation of the IHH pathway and YAP upregulated RUNX2 expression and function (Shimoyama et al., 2007). Studying the interactions of the Wnt pathway, IHH, and YAP in the context of MSC hypertrophy will shed light on the network that dictates hypertrophy. Recently, iPSCs-derived multipotent cells displayed lower hypertrophy potential than MSCs after chondroinduction (Diederichs et al., 2019a). However, the underlying mechanism is unknown. Comparison of these two types of cells through an omics-based method, such as RNA-Sequencing, may enable the identification of new molecules that dictate the hypertrophy process.

Although the similarity of these two processes requires further investigation, knowledge from cells within the epiphyseal growth plate should still be informative for future study of *in vitro* hypertrophy of chondroinduced MSCs. The comparison between resting and hypertrophic chondrocytes in the growth plate confirmed that the cells in the resting zone are maintained in a Wnt-inhibitory environment (Hallett et al., 2021).

The use of spatial transcriptomics and/or proteomics in the growth plate can precisely define gene and protein levels in these 2 cell types. These findings can potentially identify the molecules involved in the network that governs hypertrophy. Presently, similar studies have not been reported.

Another strategy is to mine publicly available data, in particular omics data. Current studies associated with these datasets only investigated a small fraction of data points. We can use machine learning and other bioinformatic strategies to analyze large quantities of data that may assist in improved mapping of the hypertrophic development network. For example, Ochsner et al. developed a web knowledgebase, which incorporates the nodes of signaling pathways (receptors, enzymes, transcription factors and co-nodes) and their cognate bioactive small molecules (Ochsner et al., 2019). This tool enabled the prediction of pathway node-gene target transcriptional regulatory relationships.

Currently, extensive Wnt inhibitors that target different components in this pathway have been reported. Screening Wnt inhibitors that displayed high hypertrophy-suppressing potential with low side-effect is also critical. Recently, SM04690, a kind of small-molecule Wnt pathway inhibitor, was shown to be effective in treating OA. In particular, SM04690 may protect chondrocytes from dedifferentiation and hypertrophic chondrocyte conversion (Deshmukh et al., 2018). Given the demonstrated safety of SM04690, this new type of Wnt inhibitor is ready for clinical trials to enhance MSCs-based therapy for repairing articular cartilage in humans.

Lastly, the Wnt pathway is critical in maintaining cell function and viability. For example, long-term treatment with PKF, a Wnt inhibitor, induced cell apoptosis in MSCs-derived cartilage (Huang et al., 2018). Therefore, the timing and duration of suppressing Wnt/β-catenin pathway to reduce hypertrophy require careful investigation.
